# Health and economic growth: Evidence from dynamic panel data of 143 years

**DOI:** 10.1371/journal.pone.0204940

**Published:** 2018-10-17

**Authors:** Rajesh Sharma

**Affiliations:** University School of Management and Entrepreneurship, Delhi Technological University, Delhi, India; Brandeis University, UNITED STATES

## Abstract

This paper re-examines health-growth relationship using an unbalanced panel of 17 advanced economies for the period 1870–2013 and employs panel generalised method of moments estimator that takes care of endogeneity issues, which arise due to reverse causality. We utilise macroeconomic data corresponding to inflation, government expenditure, trade and schooling in sample countries that takes care of omitted variable bias in growth regression. With alternate model specifications, we show that population health proxied by life expectancy exert a positive and significant effect on both real income per capita as well as growth. Our results are in conformity with the existing empirical evidence on the relationship between health and economic growth, they, however, are more robust due to the presence of long-term data, appropriate econometric procedure and alternate model specifications. We also show a strong role of endogeneity in driving standard results in growth empirics. In addition to life expectancy, other constituent of human capital, education proxied by schooling is also positively associated with real per capita income. Policy implication that follows from this paper is that per capita income can be boosted through focussed policy attention on population health. The results, however, posit differing policy implications for advanced and developing economies.

## Introduction

**Fig 1:**
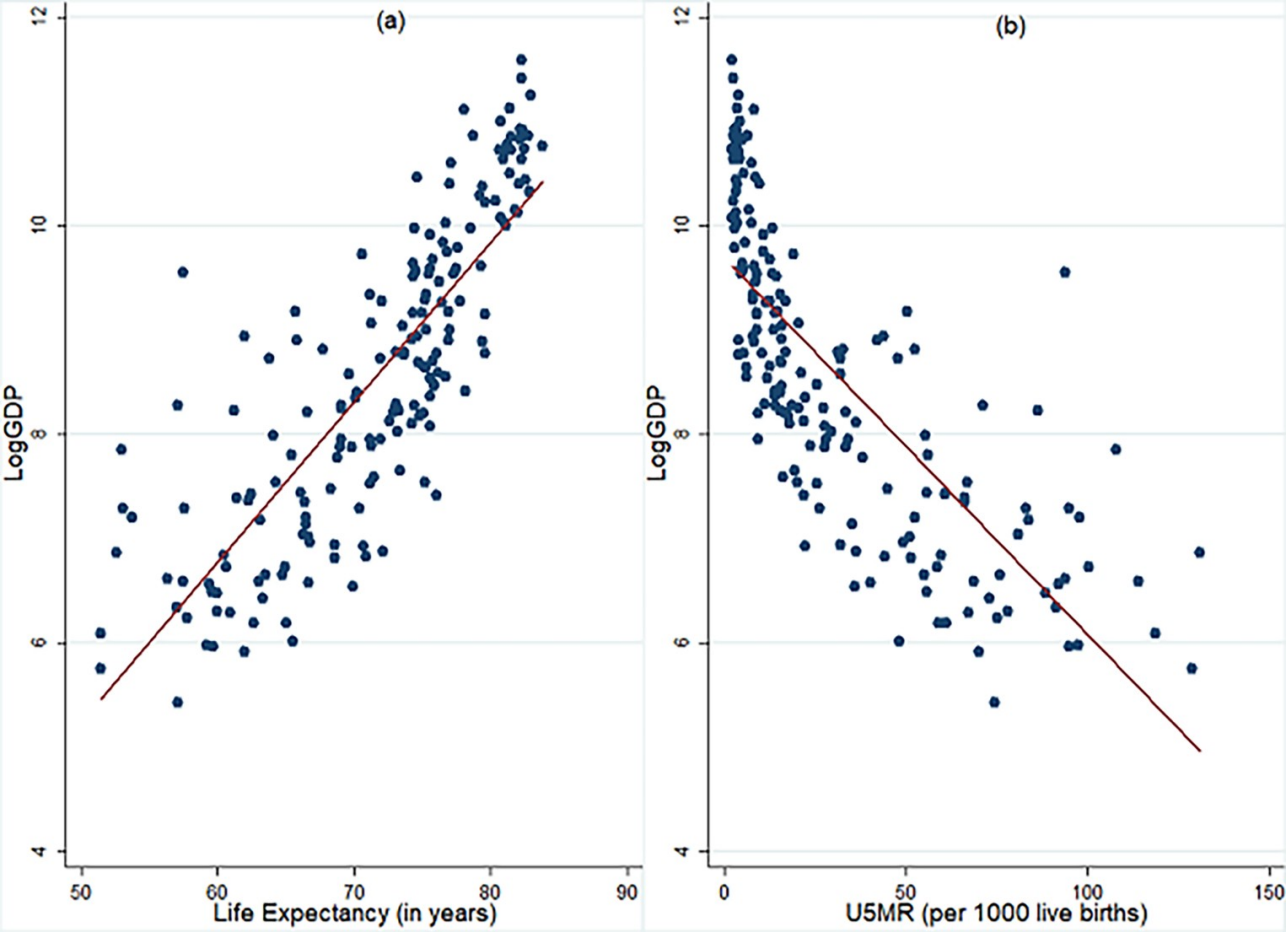
Relation between income per capita and indicators of population health. **a) LogGDP vs Life Expectancy b) LogGDP vs U5MR.** The data pertains to 193 countries for 2015 and is procured from World Development Indicators (WDI) database of World Bank. LE: Life Expectancy at birth (in years). U5MR: Under-five mortality rate is the probability per 1,000 that a new-born child will die before reaching age five, if subject to age-specific mortality rates of the specified year. LogGDP: Natural Logarithm of real GDP per capita at constant 2010 USD.

Researchers and policymakers strive hard to identify factors that influence economic growth to aid policymaking and implementation. Human capital made inroads into growth framework following endogenous growth revolution and is identified as one of the most important contributors to economic growth [1–3]. Recently, there are more evidences to the positive effect of health and healthcare investments in economic growth [4–5] and the importance of human capital for economic growth is re-emphasised by the World Bank [6–7]. Health and education are the most important constituents of human capital. Focussing on health, Fig 1A and 1B illustrates that rich countries are healthier when compared to poor countries, which gives rise to a question: whether rich countries are healthier because they are rich, or they are rich because they are healthier?

This question is analysed using a variety of population health indicators such as life expectancy [8], adult survival rate [9], child mortality [10] and adult height [11]. Theoretically, a healthy workforce contributes to greater economic output due to increased longevity as well as due to lesser number of working days lost due to ill-health. Moreover, a healthy workforce can produce more ideas and thereby contributes towards improved productivity of existing capital [1]. However, increased longevity may depress per capita income due to increased population and thin spreading of existing resources over large population [12]. Moreover, improved health may lead to substitution in favour of leisure as the same amount of income can be earned through lesser number of days of work. Which channel works, or which force dominates? Is increased longevity sufficient to compensate for its depressing effect or is there a plateau beyond which the effect of improved health starts diminishing, or it continues to provide productivity gains over infinite horizon as predicted in AK type endogenous growth models? In this paper, we aim to provide answers to these questions by re-examining health-growth relationship using a data-set of 17 advanced economies for the period 1870–2013. While previous studies were limited by data availability as they primarily relied on World Health Organisation (WHO) data-set for population health and Penn World Table (PWT) dataset for economic indicators, this paper draws strength from availability of 143 years of macroeconomic data that allows us to examine long-term relationship between economic growth and population health proxied by life expectancy. Working on a larger canvas of data is expected to unveil better insights into the long-term relationship between population health and economic growth.

The data corresponding to per capita income is made available for long time series by the Maddison project and the data pertaining to life expectancy is made available by Human Mortality Database [13] that provides data from 18^th^ century for a set of countries. However, only a bivariate analysis between life expectancy and economic growth would have resulted in biased and inconsistent estimates owing to omitted variable bias. We overcome the omitted variable bias in this paper by using historical macroeconomic data from Jorda, Schularik and Taylor ([14], JST from hereon) database which provides data for 17 advanced economies from 1870. The JST database contains data for consumer price index (CPI), merchandise trade, government expenditure and investment to GDP ratio, all of these factors have been observed to be robust determinants of economic growth.

In addition to resolving omitted variable concerns, we also take care of endogeneity issue in our estimation framework. This is because, growth determinants such as life expectancy or inflation may be affected by per capita income, thereby resulting in biased estimates due to reverse causality concerns. Concerning endogeneity in growth regression Caselli et al. [15] writes “*At a more abstract level*, *we wonder whether the very notion of exogenous variables is at all useful in a growth framework*.” To control for endogeneity issues arising out of reverse causality from income to life expectancy, we apply panel generalised method of moments (GMM) technique [16–18]. The advantage with panel GMM methodologies is that we do not require separate instrument variable for each endogenous variable, rather, we can use internal instruments, which are made available by the panel nature of the dataset. In this paper, we also make a distinction between levels *versus* growth effect. Generally, researchers consider either real per capita income or its growth rate as the dependent variable to identify whether a given macroeconomic, demographic or any other factor influences levels of per capita income or its growth rate. In this paper, we consider both variables as dependent variables separately, and identify whether population health proxied by life expectancy has significant association with the levels of real per capita income and growth rate.

The main contribution of the paper is to assemble data on life expectancy and per capita income for the period beginning 1870. Moreover, in order to abate omitted variable concerns in growth regression, we could also gather data of macroeconomic variables, which are found to be significant in growth regression. The presence of data-set for such a long period allows us to examine long-run relationship between health and growth which was earlier not possible due to data-set dating back to 1960, only. Our data-set covers a period of time in which countries experienced changes in both economic and demographic profile which provides us with sufficient dynamics to study health-growth relationship as well as to provide few policy implications. Second, we also employ one of the most appropriate econometric procedures that takes care of possible endogeneity concerns while examining health-growth relationship.

Rest of this paper is structured as follows. The section titled “Materials and Methods” is devoted to data and econometric methodology employed in the paper. The section titled “Results and Discussion” provides empirical results and ensuing discussion of results along with the limitations of the paper. The section titled “Concluding Remarks” sums up the paper and provides few avenues of future research.

## Materials and methods

### Data and variables

We employ an unbalanced panel of 17 advanced economies for the period 1870–2013. The sample countries are: Australia, Belgium, Canada, Denmark, Finland, France, Germany, Italy, Japan, Netherlands, Norway, Portugal, Spain, Sweden, Switzerland, United Kingdom, and United States (S1 Table provides key data for sample countries). The choice of sample countries is purely governed by data availability as long time series data exceeding a century of modern macroeconomic history is available only in these set of countries.

The level of economic development is represented by GDP per capita at purchasing power parity, expressed in natural logarithmic terms, as is commonly used in growth literature. As control variables, we include investment to GDP ratio (INVEST), average years of total schooling (SCHOOLING), total merchandise trade (exports plus imports) to GDP ratio (OPEN), INFLATION and government expenditure to GDP ratio (GOVT_EXP). The choice of control variables is dictated by growth theory in which these variables along with measures of human capital are found to be the most robust determinants of economic growth (see Table 1 for variable definitions and sources of data). All the data is averaged over 10 years to examine the long-term effects of population health on income and to take care of short-term fluctuations [15]. In growth literature, it is a general practice to work with data averaged over five years to arrive at long-term effects of growth determinants [15, 19, 20]. However, the presence of long time series data in this paper allows us to work with ten-year averaged data, which is expected to provide more robust evidence of long-term relationship between population health and economic growth.

**Table 1:** Variable description and sources of data.

Variable	Variable Description	Data Source
LogGDP	Natural Logarithm of real per capita income (at PPP prices)	JST Database^.^ (Schularik and Taylor [21] and Jorda, Schularik and Taylor [14, 22])
GROWTH	Ln (GDP_t_)- Ln (GDP_t-1_)	JST Database
INFLATION	Ln (CPI_t_)- Ln (CPI_t-1_)	JST Database
INVEST	Investment to GDP Ratio	JST Database
GOVT_EXP	Government Expenditure to GDP ratio	JST Database
OPEN	Ratio of [Exports +Imports] to GDP	JST Database
LIFE EXPECTANCY	Life Expectancy at birth (in years)	HMD Database and WDI Database*
SCHOOLING	Mean years of total schooling in adult population (15–64 years old)	Lee and Lee [23, 24]

* Data for Germany is procured from World Development Indicators which is available from 1950 whereas data from HMD was available only from 1990.

Data is averaged over 1871–1880, 1881–1890 and so on with last observation averaged over the period 2001–2013 providing us with a maximum of 14 observations per country. As schooling data is available at five-year frequency, we used data of median year as representative of the decade. For instance, data of schooling in 1875 serves as one observation in the decadal average of 1871–1880. Natural logarithm of real income per capita in 1870 serves as *initial income* for the decade 1871–1880 and so on with natural logarithm of real income per capita in 2000 serving as initial income for the period 2001–2013 (see Table 2 for descriptive statistics).

**Table 2:** Descriptive statistics.

Variable	No. of Obs.	Mean	Std. Dev	Min.	Max.
GROWTH	238	0.019	0.017	-0.045	0.087
LogGDP	238	8.613	0.896	6.669	10.333
INFLATION	238	0.049	0.175	-0.062	2.573
INVEST	225	0.184	0.060	0.052	0.356
GOVT_EXP	233	0.318	0.190	0.019	0.849
OPEN	237	0.427	0.349	0.060	2.855
LIFE EXPECTANCY	194	64.650	12.372	32.666	82.515
SCHOOLING	238	5.983	3.273	0.150	13.010

Based on ten-year averaged data. GROWTH: growth rate of real GDP per capita (at PPP prices); LogGDP: natural logarithm of real GDP per capita at PPP prices; INFLATION: calculated as difference in natural logarithm of CPI; INVEST: investment to GDP ratio; GOVT_EXP: government expenditure to GDP ratio; OPEN: total merchandise trade to GDP ratio; LIFE EXPECTANCY: life expectancy at birth; SCHOOLING: average number of total years of schooling.

#### Life expectancy as indicator of population health

We use life expectancy at birth as a proxy of population health. Although health is a multi-dimensional concept and life expectancy is one of the most widely used indicator of population health [8, 12, 25, 26], it is not without limitations. For instance, a country may have high life expectancy but majority of its population might be suffering from illness and might not be productive. For instance, in poor economies, due to poor nutritional availability during childhood, a child will be lesser productive in performing tasks in future life expectancy may not be high because of available medical care [9]. A better measure may be health adjusted life expectancy (HALE) that calculates healthy years of life that a child at birth is expected to live [27]. However, data pertaining to HALE or other indicators of population health such as adult mortality rate (AMR) or child mortality (U5MR) is available only for shorter duration.

**Fig 2:**
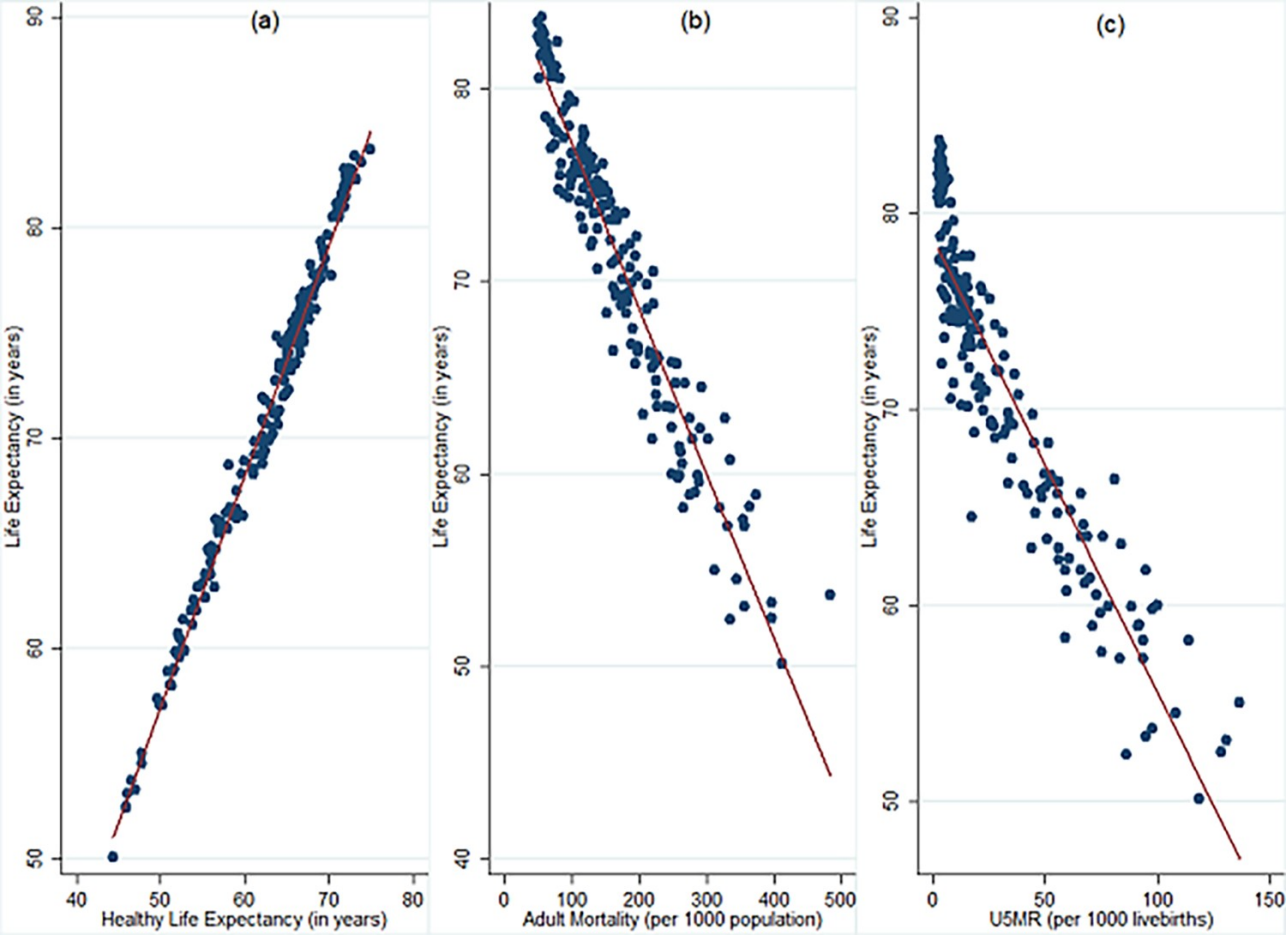
Relationship between life expectancy and other indicators of population health. **a) LE vs HALE b) LE vs AMR c) LE vs U5MR.** LE: Life Expectancy at birth (in years), HALE: Healthy Life Expectancy (at birth), AMR: Adult Mortality Rate (per 1000 population) and U5MR: Under-5 mortality Rate (per 1000 livebirths). The data pertains to 183 countries in 2015 and is procured from WHO mortality and global health estimates database.

We employ life expectancy at birth as the indicator of population health for two reasons. **First**, the data pertaining to life expectancy extends back to 19^th^ century which enables a better empirical examination between population health and economic growth. **Second**, life expectancy has a strong, albeit not perfect, correlation with most other indicators of population health. For instance, the correlation of life expectancy at birth with healthy life expectancy at birth (HALE), adult mortality rate (AMR) and child mortality (U5MR) is 0.99, -0.96 and 0.93, respectively (See Fig 2A–2C). Therefore, life expectancy may be regarded as a good, although not perfect, representative indicator of population health (See S2 Table for data used in the figure).

### Econometric methodology

Our basic regression framework builds upon Barro-type [28] growth regression model as specified below:

$$y_{i}=\alpha +\gamma X+\varepsilon_{i}$$ (1)

Here, i = 1,2,3…………n indexes cross-sections, y_i_ is the per capita income of i^th^ cross-section, X is the vector of growth determinants and ε_i_ is the stochastic error term. Above regression specified in cross-sectional framework suffers from two main limitations. *First*, it doesn’t take care of individual heterogeneity and the assumption of identical production function may result in biased coefficient estimates of γ [15]. This happens because of ignoring country-specific fixed effects in above regression, which may be correlated with regressors, therefore, estimation of cross-sectional regression Eq (1) through ordinary least squares (OLS) leads to biased coefficient estimates [15]. *Second*, it doesn’t exploit the time dimension of the data-set. Panel estimation which relaxes the restrictive assumption of identical production function can take care of both limitations. Growth regression in panel framework is specified as below:

$$Y_{i,t}=\mu_{i}+\gamma X_{i,t}+\varepsilon_{i,t}$$ (2)

Here i = 1,2,3… … indexes country; t = 1,2,3…. indexes time; μ_i_ is the country-specific fixed effect that accounts for cross-sectional heterogeneity. In growth regression, income in previous period is also a significant determinant of income in the following period [15], therefore, it is more appropriate to specify growth regression in a dynamic panel framework as below.

$$Y_{i,t}=\mu_{i}+{\beta Y}_{i,t-1}+\gamma X_{i,t}+\varepsilon_{i,t}$$ (3)

However, estimation of Eq (3) using OLS will result in biased coefficient estimates due to correlation between fixed effects and lagged dependent variable [29]. Part of this issue can be resolved by differencing the data, which eliminates fixed effects.

$$Y_{i,t}-Y_{i,t-1}=\beta (Y_{i,t-1}-Y_{i,t-2})+{\gamma (X}_{i,t}-X_{i,t-1})+{(\varepsilon }_{i,t}-\varepsilon_{i,t-1})$$ (4)

Estimation of Eq (4) through OLS still leads to biased estimates due to correlation between (Y_i,t−1_−Y_i,t−2_) and (ε_i,t_−ε_i,t−1_). There are also endogeneity concerns due to potential reverse causality from income to growth determinants such as inflation, health and schooling. The estimation of Eq (4) using OLS fails to address the concerns imposed by endogeneity of explanatory variables. The endogeneity issue is resolved by using Y_i,t−2_ as instrument for (Y_i,t−1_−Y_i,t−2_) which is correlated with (Y_i,t−1_−Y_i,t−2_) by construction but is uncorrelated with (ε_i,t_−ε_i,t−1_) provided that new error (ε_i,t_−ε_i,t−1_) term is serially uncorrelated (Arellano and Bond [16], Eq (4) also marks the first step in first difference GMM estimation). Therefore, following moment conditions may be exploited to arrive at the estimates of β and γ.

$$E[y_{\mathrm{it}-s}(\varepsilon_{\mathrm{it}}-\varepsilon_{\mathrm{it}-1})]=0\;\mathrm{for}\;s\geq 2,t=3,4\ldots \ldots ..T.$$ (5)

A similar instrumental variable strategy can be pursued if the explanatory variables (X’s) are treated as endogenous providing us with following moment conditions.

$$E[x_{\mathrm{it}-s}(\varepsilon_{\mathrm{it}}-\varepsilon_{\mathrm{it}-1})]=0\;\mathrm{for}\;s\geq 2,t=3,4\ldots \ldots T.$$ (6)

However, moment conditions specified in Eqs (5) and (6) result in more moment conditions than the number of parameters to be estimated, thereby results in an over-identified system of equations. Such an over-identified system of equations is solved through generalised method of moments (GMM) estimation to arrive at coefficient estimates of β and γ [16].

Model estimation using panel GMM estimators gives valid estimates, provided they pass a battery of specifications tests (Arellano and Bond [16]). *First*, validity of lagged values instrumenting for the differenced variable depends crucially on their being uncorrelated with differenced error term (ε_it_−ε_it−1_) which can be tested using Sargan test of overidentifying restrictions [30]. In this paper, we rely on the results from Hansen test of over-identifying restrictions rather than Sargan test, as the later not robust to heteroscedasticity or autocorrelation [31]. Therefore, following Roodman [31], for one-step, robust estimation, we report Hansen J statistic, which is the minimized value of two-step GMM criterion function and is robust. *Second*, instrument validity depends upon the assumption of error term being serially uncorrelated [16]. We, however, require errors to be serially uncorrelated of order two only, as by construction, errors in the differenced Eq (4) are correlated of order one i.e. (ε_it_−ε_it−1_) is correlated with (ε_it−1_−ε_it−2_). Therefore, we just require errors to be uncorrelated of order 2 which is tested using Arellano and Bond test [16].

## Results and discussion

### Preliminary observations

**Fig 3:**
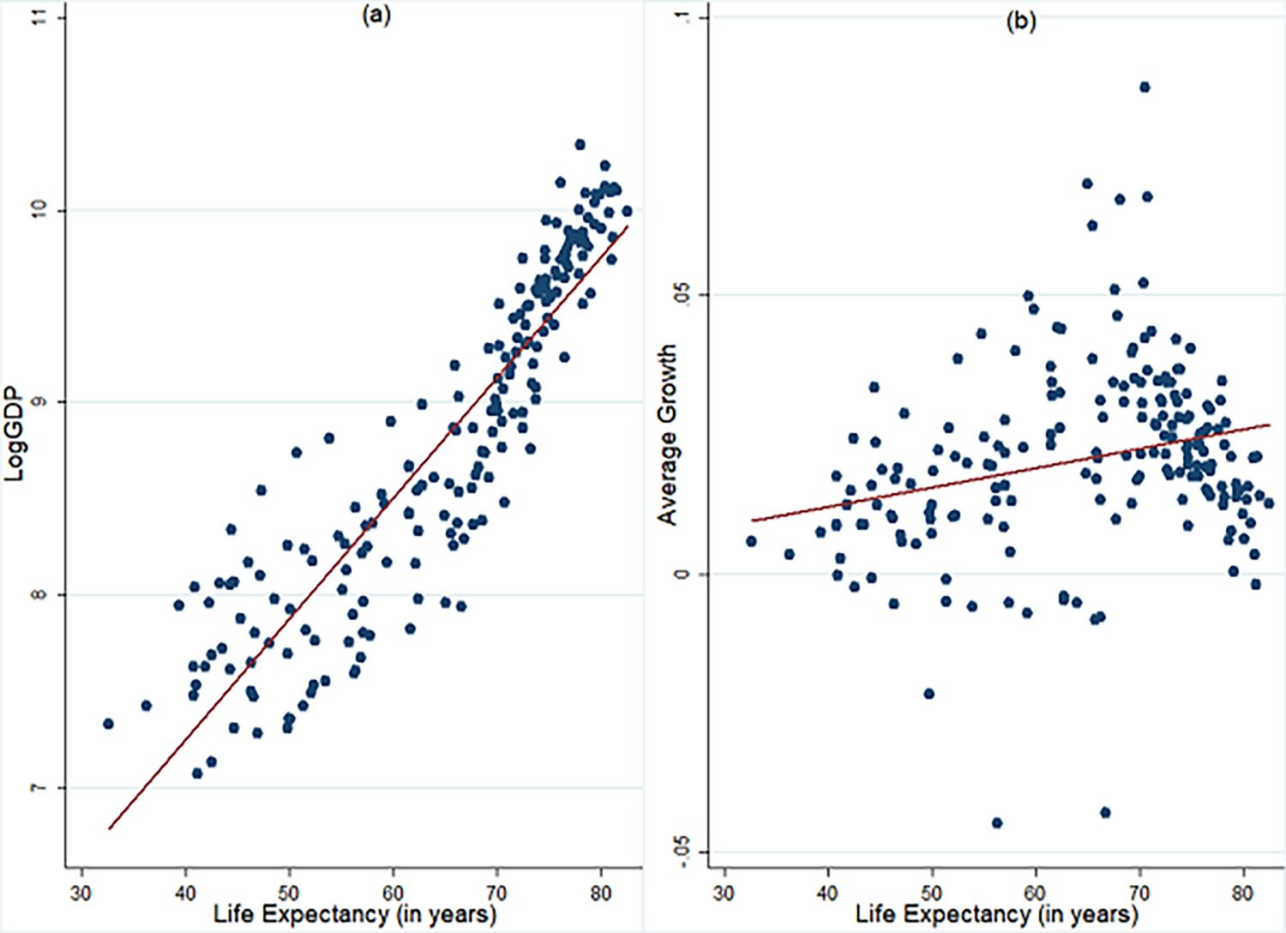
Relationship between average life expectancy and per capita income, growth. **a) Scatter plot between LogGDP and Life Expectancy b) Scatter plot between Growth and Life Expectancy.** LogGDP is the natural logarithm of real per capita income (at PPP prices). Growth: Growth rate of real per capita income calculated as difference of natural logarithm of real per capita income. Life Expectancy: Life Expectancy at Birth (in years). The data presented here is ten-year averaged.

Fig 3A and 3B presents bivariate relation between life expectancy and per capita income as well as its growth for sample countries. Life expectancy exhibits positive relation with both natural logarithm of per capita income (LogGDP) and its growth. The positive relation between life expectancy and per capita income seems to be more robust when compared to that between life expectancy and growth.

**Fig 4:**
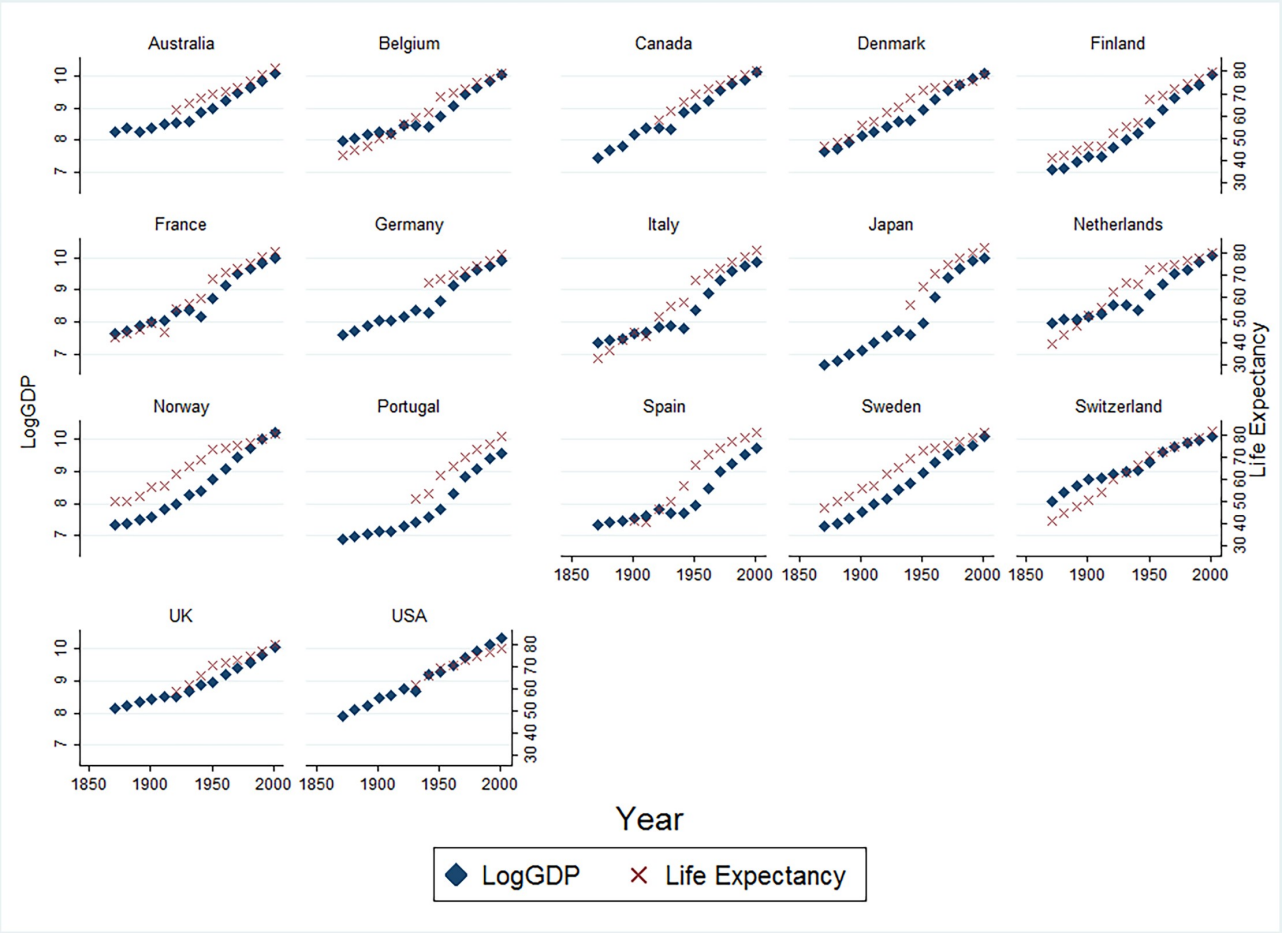
Movement of life expectancy and per capita income: By sample country. The Data pertains to 17 advanced economies in the sample. LogGDP: Natural logarithm of per capita GDP (at PPP prices).

To gain a further visual traction, Fig 4 depicts movement of LogGDP and life expectancy for each individual country in the sample which again shows a positive relation between life expectancy and per capita income.

#### Pairwise correlation

Before investigating the empirical relation between health and economic growth, it is instructive to examine the pairwise correlation between explanatory variables to be used in the regression set-up which helps in checking whether the regression results are distorted by perfect multicollinearity in regression or not. Table 3 illustrates that we can rule out the evidence of perfect multicollinearity between explanatory variables except for a high correlation (0.87), albeit not perfect, between the two components of human capital i.e. life expectancy and schooling. We will take up this issue in panel GMM estimation.

**Table 3:** Correlation matrix.

	GROWTH	LogGDP	INFLATION	INVEST	GOVT_EXP	OPEN	LIFE EXPECTANCY	SCHOOLING
GROWTH	1							
LogGDP	0.1778	1						
INFLATION	-0.0132	-0.016	1					
INVEST	0.3791	0.5985	0.0335	1				
GOVT_EXP	0.1370	0.6301	0.0460	0.4076	1			
OPEN	-0.0468	0.1821	-0.0402	0.0111	0.1486	1		
LIFE EXPECTANCY	0.2575	0.9062	0.0809	0.6875	0.6122	-0.0341	1	
SCHOOLING	0.1567	0.9133	0.0077	0.5753	0.5635	0.0899	0.8711	1

Based on 10-year averaged data. GROWTH: growth rate of real GDP per capita (at PPP prices); LogGDP: natural logarithm of real GDP per capita at PPP prices; INFLATION: calculated as difference in natural logarithm of CPI; INVEST: investment to GDP ratio; GOVT_EXP: government expenditure to GDP ratio; OPEN: total merchandise trade to GDP ratio; LIFE EXPECTANCY: life expectancy at birth; SCHOOLING: average number of total years of schooling.

### OLS results

Before moving towards panel GMM estimation, we provide results corresponding to pooled OLS (Col I, Table 4) which helps in illuminating how the coefficient estimates change by inclusion of initial income and by accounting for endogeneity of the explanatory variables. Pooled OLS results (Col I, Table 4) demonstrate that both life expectancy and schooling enter the regression positively and are significant (p<0.01). This model explains 90% of the variation in the logarithm of real per capita income (LogGDP). The other control variables such as openness (OPEN) and government expenditure (GOVT_EXP) enter the regression positively, while being statistically significant at 1% level. As discussed previously, this regression produces biased estimates as cross-sectional heterogeneity is not being accounted for in this. However, when we add fixed effects in regression framework (Col II of Table 4), new model explains 95% of variation in real per capita income (LogGDP) and the standard errors get smaller in the case of all explanatory variables. The measures of human capital, life expectancy and schooling still exert positive and statistically significant influence on per capita income (p<0.01), the coefficient size on life expectancy, however, is slightly diminished. We also observe that inflation exerts significant and negative influence on per capita income and the investment to GDP ratio (INVEST) is positively associated with per capita income (LogGDP). In column III-IV of Table 4, we replaced the dependent variable to economic growth and regressed it over the same set of explanatory variables as in column I-II. Observe that coefficient sign and statistical significance remains unchanged in most of the variables except schooling which enters the pooled OLS and fixed effect regression negatively and government expenditure no longer remains statistically significant(p>0.1). Even after adding fixed effects in growth regression, the model explains only 33% variation in growth of real per capita income as against 95% variation explained by these factors with per capita income as the dependent variable. One striking observation is that schooling exerts negative and statistically significant influence on growth of per capita income (Col III-IV, Table 4). The result seems to be at odds with the conventional wisdom of positive association between human capital and economic growth. However, such kind of anomalous observation had also been experienced previously in which human capital proxied by schooling was found to be either statistically insignificant or negative in growth regression [3, 19, 32]. As mentioned in Islam [19], the reason lies in the theoretical variable H (human capital) employed in the production function and the actual variable used in growth regression. As years of schooling may not fully account for the quality of education, an imperfect proxy for education may, therefore, manifests in the inappropriate coefficient sign or significance in growth regression.

**Table 4:** OLS results.

	I	II	III	IV
	LogGDP as dependent variable		Growth as dependent variable	
INFLATION	-0.6315	-0.9329***	-0.0686**	-0.0787**
	(0.4623)	(0.2655)	(0.0279)	(0.0330)
INVEST	0.0297	1.2533***	0.1114***	0.1278***
	(0.5929)	(0.4293)	(0.0306)	(0.0372)
GOVT_EXP	0.4040***	0.5903***	0.0005	0.0126
	(0.1470)	(0.1967)	(0.0066)	(0.0102)
OPEN	0.1883***	0.2480***	-0.0040**	0.0010
	(0.0437)	(0.0662)	(0.0019)	(0.0038)
LIFE EXPECTANCY	0.0317***	0.0223***	0.0006***	0.0008***
	(0.0053)	(0.0037)	(0.0002)	(0.0003)
SCHOOLING	0.1261***	0.1505***	-0.0028***	-0.0048***
	(0.0140)	(0.0151)	(0.0007)	(0.0009)
Intercept	5.6947***	5.7208***	-0.0176**	-0.0262**
	(0.2100)	(0.1381)	(0.0076)	(0.0085)
Fixed Effects	No	Yes	No	Yes
No. of Obs.	185	185	Yes	Yes
R^2^	0.90	0.95	0.27	0.33

Dependent variable is natural logarithm of real GDP per capita at PPP prices for column I-II and growth rate of real GDP per capita (at PPP prices) for column III-IV. The explanatory variables are: INFLATION: difference in natural logarithm of CPI; INVEST: investment to GDP ratio; GOVT_EXP: government expenditure to GDP ratio; LIFE EXPECTANCY: life expectancy at birth; OPEN: total merchandise trade to GDP ratio; SCHOOLING: average number of total years of schooling. Standard errors are heteroscedasticity corrected robust errors and are presented in parentheses.

*/**/*** denote statistical significance at 10/5/1 percent, respectively.

Although not perfect, high correlation between schooling and life expectancy may be distorting the coefficient estimates, the results corresponding to OLS excluding schooling are presented in S3 Table, which again demonstrates that life expectancy still exerts positive and statistically significant influence on real per capita income as well as its growth.

### Panel GMM results: Effect of health on per capita income

Results presented in Table 4 provide evidence of significant effect of population health on economic growth. The estimates, however, cast few doubts on appropriateness of model specification as rich countries are healthier than poorer ones i.e. there may be evidence of reverse causality from income to health. Moreover, some of the effects picked up by the explanatory variables may be the underlying ones of lagged income as economic growth may sustain due to growth momentum. Therefore, appropriate model of economic growth must account for the effects of lagged income as well as must take care of endogeneity issues that arise due to reverse causality concerns. Therefore, we estimate the Eq (3) using first difference GMM in which all the variables are considered endogenous. The model may suffer from instrument proliferation issue, therefore, in order to reduce the instrument count, we use only two lags as instruments and collapse the instruments as suggested by Roodman [31, 33].

Table 5 shows that lagged income is positively associated with current income which provides evidence of persistence in the dependent variable i.e. real income per capita (Col I-II). The effect of life expectancy on per capita income is statistically insignificant when it is treated as exogenous (p>0.1). However, the regression in which it is treated as endogenous, life expectancy exerts positive and statistically significant (p<0.05) effect on per capita income, schooling, however, is negative although statistically insignificant (p>0.1). We observe that inflation exerts negative and statistically significant (p<0.01) effect on per capita income, while the effect of trade openness is positive although significant only at 10% level. The model diagnostic tests show that errors are serially uncorrelated of order 2 (p-value = 0.473). Hansen test has p-value of 0.440 which implies that we fail to reject null hypothesis of no overidentifying restrictions.

**Table 5:** Panel GMM results with LogGDP as dependent variable.

	First Difference GMM (LE Exogenous)	First Difference GMM (LE Endogenous)	System GMM (LE Exogenous)	System GMM (LE Endogenous)
INITIAL	0.8704***	0.7229***	0.8031***	0.7422***
	(0.0704)	(0.0829)	(0.0490)	(0.0588)
INFLATION	-0.8349***	-0.6772***	-0.7961***	-0.6773***
	(0.2229)	(0.1873)	(0.1919)	(0.1950)
INVEST	1.6645**	0.5937	1.4314***	1.0232**
	(0.7005)	(0.6453)	(0.4738)	(0.4878)
GOVT_EXP	0.2525	0.1392	0.1539	0.0325
	(0.2966)	(0.2099)	(0.1678)	(0.1615)
OPEN	0.1578**	0.2827*	0.0698*	0.0815**
	(0.0788)	(0.1678)	(0.0416)	(0.0368)
LIFE EXPECTANCY	0.0039	0.0195**	0.0014	0.0096**
	(0.0064)	(0.00997)	(0.0041)	(0.0040)
SCHOOLING	-0.0034	-0.0129	0.0311	0.0244*
	(0.0244)	(0.0338)	(0.0194)	(0.0146)
No. of Obs.	166	166	185	185
No. of Instruments	13	14	13	22
Hansen Test p-value	0.420	0.440	0.395	0.544
AR (2) test p-value	0.339	0.473	0.660	0.892

Dependent variable LogGDP: natural logarithm of real GDP per capita at PPP prices. The explanatory variables are: INITIAL: natural logarithm of initial real per capita income (at PPP prices) at period t-1; INFLATION: difference in natural logarithm of CPI; INVEST: investment to GDP ratio; GOVT_EXP: government expenditure to GDP ratio; LIFE EXPECTANCY: life expectancy at birth; OPEN: total merchandise trade to GDP ratio; SCHOOLING: average number of total years of schooling. Standard errors are heteroscedasticity corrected robust errors and presented in parentheses.

*/**/*** denote statistical significance at 10/5/1 percent respectively. Null hypothesis of AR (2) test: errors are serially uncorrelated at order 2. Null hypothesis of Hansen test: there are no overidentifying restrictions.

The model estimated using first difference GMM model and presented in column 1-II of Table 5 may suffer from weak instrumental bias problems [18, 34]. Therefore, we also estimate the growth regression using system GMM which provides lesser biased coefficient estimates [18, 34, 35]. The system GMM approach [18] combines in a system, a regression in differences with regression in levels with the aim that additional moment conditions would be generated increasing the efficiency of resulting estimators. The idea of the system GMM is to estimate level equation and first difference equation simultaneously, and suggests using lags as instrument for differenced equation and differenced variables as instrument for the level equation. Thus, additional moment conditions for second part of the system (level equation) are:

$$E{[\Delta y}_{it-i}(\mu_{i}+\varepsilon_{it})]=0$$ (7)

$$E{[\Delta X}_{it-i}(\mu_{i}+\varepsilon_{it})]=0$$ (8)

In system GMM, additional moment conditions specified by Eqs (7) and (8) along with those presented in Eqs (5) and (6) generate consistent and relatively more efficient estimates compared to those obtained in the first difference GMM model.

We observe that in system GMM results (Col III-IV of Table 5), the standard errors are substantially reduced in most of the variables. With system GMM results too, we observe that life expectancy exerts positive effect on per capita income when it is treated as endogenous which is justifiable given the reverse causality concerns mentioned previously. In system GMM results in which the life expectancy is considered endogenous, schooling also exerts positive and significant influence on per capita income (p<0.1). The other explanatory variables such as inflation, investment to GDP ratio (INVEST), openness and government expenditure to GDP ratio (GOVT_EXP) have similar sign and statistical significance as observed before (Table 4 and Col I-II of Table 5). Model specification tests also demonstrate that moment conditions employed in the system GMM are appropriate–errors are uncorrelated of order 2 (AB test p-value >0.1) and there are no overidentifying restrictions (Hansen test p-value > 0.1).

### Panel GMM results: Effect of health on economic growth

In addition to investigating the effect of health on per capita income, we also examine the effect of life expectancy on economic growth using panel GMM regression (Table 6). In regression Eq (3), we replace LogGDP with growth in per capita income as the dependent variable. We obtain few results similar to those obtained with real per capita income (LogGDP) as the dependent variable such as positive and significant effect of life expectancy (p<0.05) on economic growth in both first difference and system GMM regression. Initial income per capita (INITIAL) has negative effect on subsequent growth which is in accordance with convergence hypothesis (Caselli et al [15]). Inflation has statistically significant negative influence on economic growth with both GMM estimators and investment to GDP ratio is significant (p<0.1) only in system GMM results (Col II, Table 6). In both the models, the model diagnostic tests show that errors are serially uncorrelated of order 2 (p>0.1). We also fail to reject the null hypothesis of no overidentifying restriction as the Hansen-J statistic has p-value greater than 0.10.

**Table 6:** Panel GMM results with growth as dependent variable.

	First Difference GMM	System GMM Estimation
INITIAL	-0.0407***	-0.0296***
	(0.0156)	(0.0105)
INFLATION	-0.0841*	-0.0937***
	(0.0491)	(0.0334)
INVEST	-0.0327	0.1280*
	(0.0896)	(0.0739)
GOVT_EXP	0.0248	0.0057
	(0.0390)	(0.0168)
OPEN	0.0568*	0.0090
	(0.0337)	(0.0058)
LIFE EXPECTANCY	0.0049**	0.0018**
	(0.0020)	(0.0007)
SCHOOLING	-0.0095	-0.0001
	(0.0063)	(0.0026)
No. of Obs.	166	185
No. of Instruments	14	22
Hansen Test p-value	0.321	0.443
AR (2) test p-value	0.301	0.086

Dependent variable: GROWTH: growth rate of real GDP per capita (at PPP prices) calculated as difference in natural logarithm of real per capita income (at PPP prices). The explanatory variables are: INITIAL: natural logarithm of initial real per capita income (at PPP prices) at time t-1; INFLATION: is calculated as difference in natural logarithm of CPI; invest: investment to GDP ratio; GOVT_EXP: government expenditure to GDP ratio; LIFE EXPECTANCY: life expectancy at birth; OPEN: total merchandise trade to GDP ratio; SCHOOLING: average number of total years of schooling. Standard errors are heteroscedasticity corrected robust errors and are presented in parentheses.

*/**/*** denote statistical significance at 10/5/1 percent, respectively. Null hypothesis of AR (2) test: errors are serially uncorrelated at order 2. Null hypothesis of Hansen test: there are no overidentifying restrictions.

### Panel GMM results: Effect of health excluding schooling

From Table 3, we observed high correlation between life expectancy and schooling. Therefore, to extract more robust estimates of life expectancy on per capita income and economic growth, we re-ran regression (3) using first difference and system GMM regression excluding schooling as one of the explanatory variables (Table 7). Comparing results of regression including schooling (Tables 5 and 6) and with those after excluding it (Table 7), the coefficient on life expectancy is still significant and there is not much change in its coefficient estimate. For instance, in system GMM regression, the effect of life expectancy on per capita income changes only from 0.0096 (Col IV, Table 5) to 0.0086 (Col II, Table 7) and on growth it changes marginally from 0.0018 (Col II, Table 6) to 0.0016 (Col IV, Table 7).

**Table 7:** Panel GMM results excluding schooling.

	First Difference GMM	System GMM	First Difference GMM	System GMM
INITIAL	0.6194***	0.8274***	-0.0596***	-0.0292***
	(0.1299)	(0.0395)	(0.0197)	(0.0067)
INFLATION	-0.6256***	-0.7611***	-0.0914**	-0.0978**
	(0.2343)	(0.1716)	(0.0463)	(0.0390)
INVEST	0.2291	1.0962**	-0.0231	0.1397*
	(0.8185)	(0.4627)	(0.1097)	(0.0819)
GOVT_EXP	0.2133	0.0740	0.0276	0.0062
	(0.2401)	(0.1463)	(0.0365)	(0.0177)
OPEN	0.3376*	0.0856***	0.0485	0.0096**
	(0.1839)	(0.0254)	(0.0301)	(0.0043)
LIFE EXPECTANCY	0.0239**	0.0086**	0.0037**	0.0016**
	(0.0113)	(0.0039)	(0.0017)	(0.0008)
No. of Obs.	166	166	185	185
No. of Instruments	12	19	12	19
Hansen Test p-value	0.595	0.644	0.113	0.256
AR (2) test p-value	0.340	0.681	0.267	0.113

Dependent variable is natural logarithm of real GDP per capita at PPP prices for column I-II and growth rate of real GDP per capita (at PPP prices) for column III-IV. The explanatory variables are: INITIAL: natural logarithm of initial real per capita income (at PPP prices); INFLATION: is calculated as difference in natural logarithm of CPI, INVEST: investment to GDP ratio; GOVT_EXP: government expenditure to GDP ratio; LIFE EXPECTANCY: life expectancy at birth; OPEN: total merchandise trade to GDP ratio. Standard errors are heteroscedasticity corrected robust errors and presented in parentheses.

*/**/*** denote statistical significance at 10/5/1 percent, respectively. Null hypothesis of AR (2) test: errors are serially uncorrelated at order 2. Null hypothesis of Hansen test: there are no overidentifying restrictions.

## Discussion

Through alternate model specifications and econometric procedures, we found that life expectancy has positive and statistically significant effect on per capita income as well its growth. Our regression results also indicate a strong role of endogeneity in driving standard results in growth empirics [15]. What are the policy implications going forward and what developing countries can learn from the experience of developed countries? Our results implicate a scope of boosting economic growth through focussed policy attention on population health by increased investments in healthcare systems. Secondly, as shown in Fig 1, there is very strong correlation between life expectancy and other indicators of population health which implies that till the time we get a better summary indicator of population health for which data is available for longer periods, a focus on boosting longevity seems to be an optimal strategy. From S2 Table, we notice that life expectancy ranges from the lows of 50.2 and 52.4 in Sierra Leone and Angola, respectively, to 83.4 and 83.7 in Switzerland and Japan, respectively in 2015. Such low levels of life expectancy imply high child mortality, lower adult survival rate and high prevalence of disease burden in working age-group which implies a significant loss to human capital which significantly impairs its contribution to economic production. Moreover, low levels of life expectancy lead to higher fertility and consequent population growth leading to further thinning of existing resources per capita. Therefore, developing countries have a scope to boost their income levels by focussing on population health. This raises a question: how to boost population health? What are the challenges to healthcare systems in advanced economies and low-resource economies? In terms of boosting longevity, reduction in child mortality has played a crucial role in the past [36] and is expected to provide further gains in future for low- and low-middle income economies. Moreover, many of the diseases that account for premature deaths as well as a cause of morbidity are preventable as well as treatable with available low-cost interventions [37]. For instance, 19.5 million children under the age 1 did not receive the three recommended doses of Diphtheria, Tetanus and Pertussis (DTP3) in 2016, and 20.8 million children below the age 1 didn’t receive a single dose of measles-containing vaccine [38]. Lastly, if we compare general government expenditure on health (Table 8) it is found to be significantly lower in poor economies. Moreover, in the light of stagnating development assistance for health (DAH) to poor economies, there is going to be significant pressure on already constrained public resources in poor economies [39].

**Table 8:** Health expenditure and health status in country groupings.

Country Groups	Domestic general government health expenditure (% of GDP)	Out-of-pocket expenditure (% of current health expenditure)	Immunization, DPT (% of children ages 12–23 months)	Life expectancy at birth, total (years)
Low Income	1.26	40.42	77.54	62.11
Lower middle income	1.30	57.28	80.48	67.70
Upper middle income	3.34	31.56	94.42	75.14
High income	7.82	13.50	95.76	80.50

**Note**: Data is from World Development Indicators: Database of World Bank

**Fig 5:**
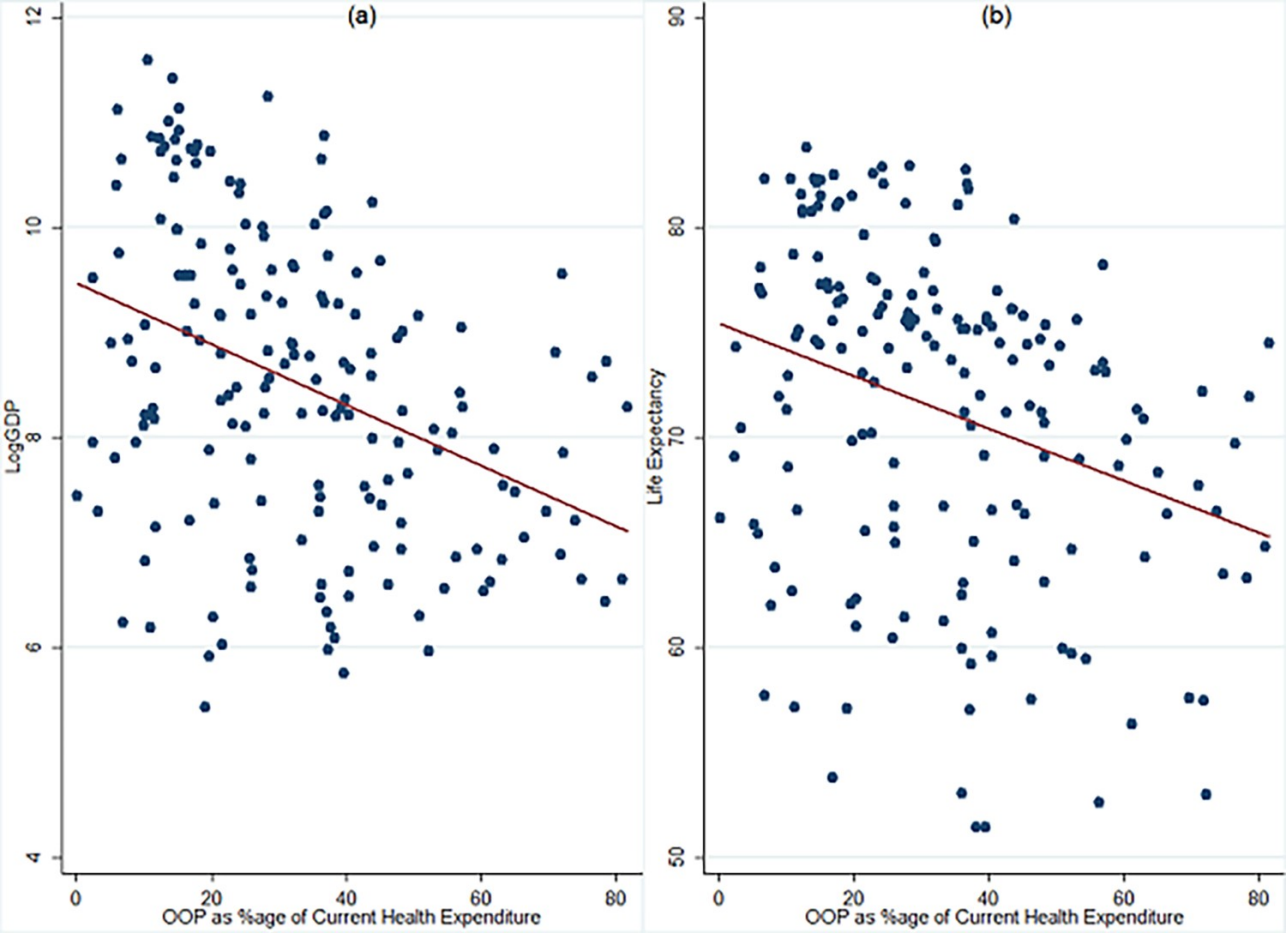
Relationship between income, life expectancy and OOP. **a) Scatter plot between LogGDP and OOP b) Scatter plot between LE and OOP.** The data pertains to 193 countries and is procured from World Development Indicators (WDI) database of World Bank. LogGDP: Natural Logarithm of real GDP per capita at constant 2010 USD. LE: Life Expectancy at Birth. OOP: Out-of-pocket expenditure as percentage of current health expenditure (CHE). Data of all variables pertains to 2015 and is procured from World Development Indicators (WDI) database of World Bank.

Table 8 also demonstrates that in the absence of insurance cover, a large chunk of health expenditure is incurred as out-of-pocket expenditures (OOP) which further pushes people below the poverty line (see Fig 5). Health expenditure is thus a significant drain on poor household’s resources and significantly impairs their human capital, which has further negative bearing on economic growth. In the light of high OOP expenditure in low and lower-middle income economies, the access to universal health coverage (UHC) is felt most urgently which is also emphasised in Sustainable Development Goal 3.8 [40].

Next question that demands urgent attention is: will life expectancy gains continue to persist and continue to provide economic gains forever? The answer to this question seems to be not clear as endogenous growth theory involving AK type of production function assume that investments in human capital continues to provide positive results forever by boosting productivity (A). However, economic rationale of diminishing marginal returns suggests that improvements in human capital may eventually lead to non-significant economic gains in future. Now, advanced economies which have witnessed substantial gains in life expectancy over last 140 years are standing on the verge of stagnating gains as most of health gains due to reduction in mortality from communicable causes have almost vanished and their place has increasingly been taken up by non-communicable diseases such as cancer, cardiovascular diseases and neurological disorders etc. [41]. The signs of non-significant gains in longevity may already be visible in developed countries (Fig 4). Secondly, there may be evidence of diminishing marginal returns to improved longevity evidenced by Global Burden of Disease study 2016 [42, 43], which shows that longer lives may not be translating into healthy longer lives. This is because we may have found solutions to many of leading communicable causes of death but the burden of non-communicable diseases such as cardiovascular diseases, diabetes, and neoplasms is increasing alongwith the burden of disorders such as neurological disorders, musculoskeletal disorders and substance abuse disorders, the later may not be claiming lives but are responsible for a surge in burden due to disability. This is particularly challenging as much of this burden falls on the working age group (15–64) causing a significant loss to human capital. To summarise, the priority of public health policy in developing countries must be on boosting longevity, the advanced economies, on the other hand, must focus on ensuring healthier life to their long-living populations.

### Limitations

The main limitation of this paper is that comprehensive macroeconomic data spanning over a century is available only for a subset of advanced economies. Although, the experience of advanced economies can shed some light on health-growth nexus and has policy implications for developing countries too, still, availability of data for developing countries for long time series is expected to illuminate health-growth relationship in a more robust manner. Second, although life expectancy is strongly correlated with other indicators of population health, still it is not a perfect measure of population health [9].

## Concluding remarks

Sustainable Development Goal 3 seeks to *ensure healthy lives and promote well-being for all at all ages*. In this paper, we investigate whether population health has causal effect on per capita income and its growth or not, using an unbalanced panel of 17 advanced economies for the period 1870–2013. We use life expectancy at birth as a proxy for population health and control for endogeneity issues using panel generalised method of moments (GMM) technique [16–18]. With alternate model specifications, we demonstrated that population health has positive and significant effect on both real income per capita as well as its growth. In addition to life expectancy, other constituent of human capital, schooling is also positively associated with per capita income. Investment has positive effect on growth which is consistent with the findings of existing literature. Government expenditure, however, has either statistically insignificant (p>0.1) or positive effect on per capita income and growth which seems to be at odds with the hypothesis of crowding out effect of government expenditure on real investments and hence, must exerts negative effect on growth. Our results may be driven by the positive effect of government expenditure during great depression and recessionary times as the data-set employed in this study contains many of the recessionary episodes. This observation cannot be generalised, however, and the dynamics of government’s size and its implications on real investments and growth demand rigorous empirical scrutiny by focussing on specific components of government expenditure. Inflation exerts negative and statistically significant influence on both per capita income and its growth and seems to be consistent with the existing literature [44]. Regarding population health, our results are consistent with previous results. Our results, however, seem to be more robust as these are based on 143 years of data, appropriate econometric specification and controlling for majority of macroeconomic determinants of growth. The probable channels through which life expectancy is envisaged to foster growth is through capital accumulation and boosting productivity of existing capital. In general, improved life expectancy leads to elongation of number of working years, also evident from worldwide debates on increasing the age of retirement. Second, majority of gains in life expectancy can be attributed to reductions in child as well as adult mortality; providing for greater number of working years and hence higher level of savings (and investments), which further propels the economic growth. This longevity-saving-growth channel has been identified as a source of East Asian growth miracle [45–46]. Health itself is not only a part of human capital but also contributes to human capita such as education [47]. Improved health (measured using life expectancy) allows the working population to be more productive in terms of new ideas and innovations. This further leads to enhanced productivity of the physical capital when employed by more skilful managers in the economy. Subsequently, longer living population invest more in human capital such as education as it expects to live greater number of years [48] leading to a virtuous cycle of improved productivity and economic growth. The empirical investigation of channels through which population health affects per capita income and growth may be an important agenda for future research.

Lastly, life expectancy may have provided gains in per capita income in the past, however, due to elevating burden of NCDs, the disability adjusted life years (DALYs) burden–sum of years of life lost (YLL) due to premature death and years lived with disability (YLD) due to injury or illness–which was earlier used to be dominated by YLLs is now increasingly getting dominated by lived with disability (YLD) even in developing nations [49]. Moreover, the increasing burden of old age diseases ranging from cancers to neurological diseases as well as life style related diseases is exacting a toll on not only elderly population but also on working age-group [37]. How health system can tackle these challenges going forward? This is particularly a challenging problem for poor countries whose health system are stressed due to twin burden of communicable diseases as well as rising burden of non-communicable diseases such as cardiovascular disorders and cancers. Going forward, an analysis of economic burden or loss in GDP due to these diseases may be an agenda for future research.

## Supporting information

S1 Table Key data for sample countries. Data Source: Based on authors’ calculation on data reported in the paper.
(DOCX)

S2 Table Key indicators of population health for 183 countries. LE: Life Expectancy at Birth (in years); LE60: Life Expectancy at the age 60 (in years); HALE: Healthy Life Expectancy at Birth (in years); HALE60: Healthy Life Expectancy at the age 60 (in years); AMR: Adult Mortality Rate (per 100,000 population); U5MR: Under 5 Mortality Rate (per 1000 livebirths).
(DOCX)

S3 Table Pooled OLS results excluding schooling as explanatory variable. Dependent variable is natural logarithm of real GDP per capita at PPP prices for column I-II and growth rate of real GDP per capita (at PPP prices) for column III-IV. The explanatory variables are: INFLATION: difference in natural logarithm of CPI; INVEST: investment to GDP ratio; GOVT_EXP: government expenditure to GDP ratio; LIFE EXPECTANCY: life expectancy at birth; OPEN: total merchandise trade to GDP ratio; SCHOOLING: average number of total years of schooling. Standard errors are heteroscedasticity corrected robust errors and are presented in parentheses. */**/*** denote statistical significance at 10/5/1 percent, respectively.
(DOCX)

S4 Table Pooled OLS results excluding life expectancy as explanatory variable. Dependent variable is natural logarithm of real GDP per capita at PPP prices for column I-II and growth rate of real GDP per capita (at PPP prices) for column III-IV. The explanatory variables are: INFLATION: difference in natural logarithm of CPI; INVEST: investment to GDP ratio; GOVT_EXP: government expenditure to GDP ratio; OPEN: total merchandise trade to GDP ratio; SCHOOLING: average number of total years of schooling. Standard errors are heteroscedasticity corrected robust errors and are presented in parentheses. */**/*** denote statistical significance at 10/5/1 percent, respectively.
(DOCX)

S5 Table Panel GMM results excluding schooling as explanatory variable. Dependent variable is natural logarithm of real GDP per capita at PPP prices for column I-II and growth rate of real GDP per capita (at PPP prices) for column III-IV. The explanatory variables are: INITIAL: natural logarithm of initial real per capita income (at PPP prices); INFLATION: calculated as difference in natural logarithm of CPI; INVEST: investment to GDP ratio; GOVT_EXP: government expenditure to GDP ratio; LIFE EXPECTANCY: life expectancy at birth; OPEN: total merchandise trade to GDP ratio. Standard errors are heteroscedasticity corrected robust errors and are presented in parentheses. */**/*** denote statistical significance at 10/5/1 percent, respectively. Null hypothesis of AR (2) test: errors are serially uncorrelated of order 2. Null hypothesis of Hansen test: there are no overidentifying restrictions.
(DOCX)
