# Supplementary material for: Health and economic growth: Evidence from dynamic panel data of 143 years
Source: PLoS One. 2018 Oct 17;13(10):e0204940. doi: 10.1371/journal.pone.0204940 (PMC6192630; doi:10.1371/journal.pone.0204940)
Supplement: S1 Table — Data Source: Based on authors’ calculation on data reported in the paper. (DOCX) [file pone.0204940.s001.docx]

**Table S1. Key data for sample countries**

| Year | Country | OPEN | INVEST | GOVT_EXP | GROWTH | INF | LogGDP | LE | SCH |
| --- | --- | --- | --- | --- | --- | --- | --- | --- | --- |
| 1870 | Australia | 0.35 | 0.11 |  |  |  | 8.09 |  | 1.77 |
| 1921 | Australia | 0.33 | 0.18 | 0.17 | 0.03 | -0.10 | 8.50 | 60.98 |  |
| 2010 | Australia | 0.35 | 0.27 | 0.49 | 0.01 | 0.03 | 10.15 | 82.07 | 11.69 |
| 1870 | Belgium | 0.36 |  | 0.09 |  |  | 7.90 | 40.90 | 2.43 |
| 2010 | Belgium | 1.69 | 0.20 | 0.57 | 0.02 | 0.02 | 10.07 | 80.07 | 11.29 |
| 1870 | Canada | 0.39 |  | 0.09 |  |  | 7.44 |  | 2.74 |
| 1921 | Canada | 0.40 | 0.17 | 0.24 | -0.13 | -0.13 | 8.12 | 57.03 |  |
| 2010 | Canada | 0.49 | 0.24 | 0.31 | 0.02 | 0.02 | 10.12 | 81.40 | 12.74 |
| 1870 | Denmark | 0.36 | 0.12 | 0.13 |  |  | 7.60 | 45.97 | 2.46 |
| 2010 | Denmark | 0.57 | 0.17 | 0.81 | 0.01 | 0.02 | 10.07 | 79.24 | 11.97 |
| 1878 | Finland | 0.45 | 0.10 |  | -0.03 | -0.10 | 7.07 | 39.15 |  |
| 2010 | Finland | 0.56 | 0.23 | 0.51 | 0.03 | 0.02 | 10.06 | 80.01 | 10.71 |
| 1870 | France | 0.24 | 0.10 | 0.20 |  |  | 7.54 | 36.38 | 0.71 |
| 2010 | France | 0.43 | 0.19 | 0.45 | 0.01 | 0.02 | 9.97 | 81.42 | 11.34 |
| 1870 | Germany |  | 0.12 |  |  |  | 7.52 |  | 2.23 |
| 1950 | Germany | 0.18 | 0.22 | 0.27 | 0.18 | -0.06 | 8.26 | 66.79 | 7.21 |
| 2010 | Germany | 0.70 | 0.17 | 0.25 | 0.04 | 0.01 | 9.94 | 79.99 | 12.58 |
| 1872 | Italy | 0.26 | 0.08 | 0.24 | -0.02 | 0.12 | 7.30 | 29.70 |  |
| 2010 | Italy | 0.55 | 0.19 | 0.56 | 0.01 | 0.02 | 9.83 | 82.10 | 10.71 |
| 1870 | Japan |  |  |  |  |  | 6.60 |  | 0.73 |
| 1947 | Japan | 0.02 | 0.17 | 0.31 | 0.07 | 0.81 | 7.34 | 51.72 |  |
| 2010 | Japan | 0.27 | 0.20 | 0.31 | 0.05 | -0.01 | 10.00 | 83.00 | 12.44 |
| 1870 | Netherlands | 0.97 | 0.09 | 0.22 |  |  | 7.92 | 37.32 | 2.94 |
| 2010 | Netherlands | 1.11 | 0.20 | 0.56 | 0.01 | 0.01 | 10.10 | 80.82 | 11.71 |
| 1870 | Norway | 0.34 | 0.12 | 0.07 |  |  | 7.22 | 50.85 | 4.31 |
| 2010 | Norway | 0.49 | 0.25 | 0.85 | 0.00 | 0.02 | 10.24 | 81.03 | 11.65 |
| 1870 | Portugal | 0.10 |  | 0.10 |  |  | 6.88 |  | 0.27 |
| 1940 | Portugal | 0.17 |  | 0.19 | -0.08 | 0.05 | 7.39 | 51.39 | 1.65 |
| 2010 | Portugal | 0.67 | 0.21 | 0.48 | 0.01 | 0.01 | 9.57 | 79.96 | 8.71 |
| 1870 | Spain | 0.12 | 0.05 | 0.20 |  |  | 7.10 |  | 1.58 |
| 1908 | Spain | 0.18 | 0.07 | 0.18 | 0.03 | -0.03 | 7.58 | 41.33 |  |
| 2010 | Spain | 0.39 | 0.22 | 0.36 | -0.01 | 0.02 | 9.73 | 82.05 | 10.75 |
| 1870 | Sweden | 0.31 | 0.09 | 0.12 |  |  | 7.20 | 44.99 | 3.61 |
| 2010 | Sweden | 0.63 | 0.22 | 0.62 | 0.06 | 0.01 | 10.14 | 81.52 | 11.95 |
| 1876 | Switzerland |  | 0.17 | 0.02 | -0.05 | 0.08 | 8.13 | 40.05 |  |
| 2010 | Switzerland | 0.61 | 0.23 | 0.21 | 0.02 | 0.01 | 10.13 | 82.30 | 12.92 |
| 1870 | UK | 0.50 | 0.07 | 0.13 |  |  | 8.07 |  | 0.92 |
| 1922 | UK | 0.43 | 0.09 | 0.40 | 0.04 | -0.15 | 8.44 | 57.03 |  |
| 2010 | UK | 0.40 | 0.16 | 0.73 | 0.01 | 0.03 | 10.08 | 80.41 | 12.46 |
| 1870 | USA | 0.11 | 0.16 | 0.09 |  |  | 7.80 |  | 4.13 |
| 1933 | USA | 0.06 | 0.03 | 0.12 | -0.03 | -0.05 | 8.47 | 60.88 |  |
| 2010 | USA | 0.22 | 0.18 | 0.38 | 0.02 | 0.02 | 10.33 | 78.83 | 13.24 |

**Data Source:** Based on authors’ calculation on data reported in the paper. Variables are defined as: OPEN: total merchandise trade to GDP ratio; INVEST: investment to GDP ratio; GOVT_EXP: government expenditure to GDP ratio; GROWTH: growth rate of real GDP per capita (at PPP); INFLATION: is calculated as difference in natural logarithm of CPI; LogGDP: natural logarithm of real GDP per capita (at PPP prices); LE: life expectancy at birth; SCH: average number of total years of schooling.
