# Supplementary material for: Health and economic growth: Evidence from dynamic panel data of 143 years
Source: PLoS One. 2018 Oct 17;13(10):e0204940. doi: 10.1371/journal.pone.0204940 (PMC6192630; doi:10.1371/journal.pone.0204940)
Supplement: S2 Table — LE: Life Expectancy at Birth (in years); LE60: Life Expectancy at the age 60 (in years); HALE: Healthy Life Expectancy at Birth (in years); HALE60: Healthy Life Expectancy at the age 60 (in years); AMR: Adult Mortality Rate (per 100,000 population); U5MR: Under 5 Mortality Rate (per 1000 livebirths). (DOCX) [file pone.0204940.s002.docx]

**Table S2 Key Indicators of Population health for 183 countries in 2015**

| Country | LE | LE60 | HALE | HALE60 | AMR | U5MR |
| --- | --- | --- | --- | --- | --- | --- |
| Afghanistan | 60.5 | 16 | 52.3 | 11.3 | 263 | 73.2 |
| Albania | 77.8 | 21.6 | 68.8 | 16.9 | 74 | 14 |
| Algeria | 75.6 | 21.8 | 66.3 | 16.7 | 109 | 25.5 |
| Angola | 52.4 | 15.8 | 45.9 | 11.7 | 335 | 86.5 |
| Antigua and Barbuda | 76.4 | 21.7 | 67.2 | 16.6 | 130 | 8.7 |
| Argentina | 76.3 | 21.5 | 67.6 | 16.7 | 116 | 11.6 |
| Armenia | 74.8 | 19.6 | 66.9 | 15.7 | 118 | 14 |
| Australia | 82.8 | 25.5 | 71.9 | 19.6 | 59 | 3.8 |
| Austria | 81.5 | 24.1 | 72 | 19.1 | 65 | 3.6 |
| Azerbaijan | 72.7 | 18.7 | 64.7 | 14.8 | 118 | 32 |
| Bahamas | 76.1 | 22.5 | 66.6 | 17.1 | 147 | 11 |
| Bahrain | 76.9 | 19.7 | 67 | 14.9 | 69 | 7.7 |
| Bangladesh | 71.8 | 19 | 62.4 | 14 | 129 | 36.3 |
| Barbados | 75.5 | 19.8 | 66.8 | 15.5 | 98 | 12.6 |
| Belarus | 72.3 | 18.8 | 65.2 | 15.4 | 196 | 4 |
| Belgium | 81.1 | 24.1 | 71.1 | 18.6 | 74 | 4 |
| Belize | 70.1 | 17.1 | 62.2 | 13.1 | 175 | 15.6 |
| Benin | 60 | 15.7 | 52.5 | 11.8 | 249 | 100.3 |
| Bhutan | 69.8 | 20.5 | 61.2 | 15.2 | 211 | 33.6 |
| Bolivia | 70.7 | 21.3 | 62.5 | 16.7 | 186 | 38.2 |
| Bosnia and Herzegovina | 77.4 | 20.6 | 68.6 | 16.1 | 88 | 6.1 |
| Botswana | 65.7 | 17.2 | 56.9 | 12.8 | 256 | 42.1 |
| Brazil | 75 | 21.7 | 65.5 | 16.2 | 142 | 15.7 |
| Brunei Darussalam | 77.7 | 21.3 | 70.3 | 17.5 | 78 | 10 |
| Bulgaria | 74.5 | 19.6 | 66.4 | 15.5 | 137 | 8.2 |
| Burkina Faso | 59.9 | 15.4 | 52.6 | 11.5 | 260 | 88.5 |
| Burundi | 59.6 | 16.5 | 52.2 | 12.2 | 288 | 74.6 |
| Cabo Verde | 73.3 | 18.9 | 64.2 | 14.2 | 114 | 22.2 |
| Cambodia | 68.7 | 17.3 | 58.1 | 10.5 | 174 | 32 |
| Cameroon | 57.3 | 16.5 | 50.3 | 12.4 | 357 | 83.3 |
| Canada | 82.2 | 25 | 72.3 | 19.7 | 64 | 5.1 |
| Central African Republic | 52.5 | 16.1 | 45.9 | 11.9 | 397 | 128.8 |
| Chad | 53.1 | 15.8 | 46.1 | 11.2 | 356 | 130.9 |
| Chile | 80.5 | 24.4 | 70.5 | 18.7 | 82 | 8.4 |
| China | 76.1 | 19.7 | 68.5 | 15.9 | 85 | 10.7 |
| Colombia | 74.8 | 21.7 | 65.2 | 16.4 | 143 | 15.8 |
| Comoros | 63.5 | 16.3 | 55.9 | 12.2 | 227 | 75.8 |
| Congo | 64.7 | 17.9 | 56.6 | 13.7 | 267 | 55.4 |
| Costa Rica | 79.6 | 24.2 | 69.8 | 18.6 | 95 | 9.1 |
| Côte d’Ivoire | 53.3 | 14.4 | 47 | 10.9 | 397 | 95.1 |
| Croatia | 78 | 21.5 | 69.4 | 17 | 95 | 4.8 |
| Cuba | 79.1 | 22.8 | 69.2 | 17.4 | 92 | 5.6 |
| Cyprus | 80.5 | 22.5 | 71.3 | 17.8 | 52 | 2.7 |
| Czech Republic | 78.8 | 22 | 69.4 | 16.9 | 86 | 3.2 |
| Democratic Republic of the Congo | 59.8 | 16.8 | 51.8 | 12.5 | 258 | 97.6 |
| Denmark | 80.6 | 23.3 | 71.2 | 18.3 | 71 | 4.3 |
| Djibouti | 63.5 | 17.6 | 55.8 | 13.2 | 241 | 66.2 |
| Dominican Republic | 73.9 | 22 | 65.1 | 16.8 | 152 | 31.5 |
| Ecuador | 76.2 | 22.7 | 67 | 17.5 | 118 | 21.5 |
| Egypt | 70.9 | 17.4 | 62.2 | 12.8 | 159 | 23.7 |
| El Salvador | 73.5 | 21.9 | 64.1 | 16.3 | 178 | 15.5 |
| Equatorial Guinea | 58.2 | 16.8 | 51.3 | 12.7 | 320 | 94 |
| Eritrea | 64.7 | 15.6 | 55.7 | 11 | 255 | 45.9 |
| Estonia | 77.6 | 22 | 69 | 17.6 | 119 | 3.1 |
| Ethiopia | 64.8 | 17.8 | 56.1 | 12.6 | 225 | 61.3 |
| Fiji | 69.9 | 17.2 | 62.9 | 13.6 | 188 | 22.5 |
| Finland | 81.1 | 24.1 | 71 | 18.5 | 76 | 2.4 |
| France | 82.4 | 25.7 | 72.6 | 20.3 | 78 | 3.9 |
| Gabon | 66 | 18.2 | 57.2 | 13.7 | 229 | 50.6 |
| Gambia | 61.1 | 15.3 | 53.8 | 11.6 | 262 | 67.5 |
| Georgia | 74.4 | 19.4 | 66.4 | 15.5 | 129 | 11.4 |
| Germany | 81 | 23.7 | 71.3 | 18.6 | 68 | 3.9 |
| Ghana | 62.4 | 15.9 | 55.3 | 12.3 | 249 | 61 |
| Greece | 81 | 24 | 71.9 | 19.1 | 72 | 3.8 |
| Grenada | 73.6 | 19 | 65 | 14.6 | 142 | 15.8 |
| Guatemala | 71.9 | 21.1 | 62.2 | 15.2 | 186 | 29.5 |
| Guinea | 59 | 16.7 | 51.7 | 12.4 | 284 | 92 |
| Guinea-Bissau | 58.9 | 15.1 | 51.5 | 11.2 | 275 | 91.3 |
| Guyana | 66.2 | 16.1 | 59 | 12.7 | 215 | 33.4 |
| Haiti | 63.5 | 17.6 | 55.4 | 13.1 | 240 | 68.9 |
| Honduras | 74.6 | 22.3 | 64.9 | 16.6 | 147 | 19.4 |
| Hungary | 75.8 | 20.3 | 67.4 | 15.8 | 134 | 5.4 |
| Iceland | 82.7 | 24.7 | 72.7 | 19.3 | 49 | 2.2 |
| India | 68.3 | 17.9 | 59.6 | 13.3 | 181 | 45.2 |
| Indonesia | 69.1 | 16.6 | 62.1 | 13.1 | 176 | 27.3 |
| Iran | 75.5 | 19.6 | 66.5 | 15.2 | 83 | 15.7 |
| Iraq | 68.9 | 18.3 | 60 | 13.4 | 182 | 32.2 |
| Ireland | 81.4 | 24.1 | 71.5 | 18.9 | 64 | 3.7 |
| Israel | 82.5 | 25 | 72.8 | 19.5 | 58 | 3.8 |
| Italy | 82.7 | 25.1 | 72.8 | 19.9 | 56 | 3.4 |
| Jamaica | 76.2 | 22.4 | 67 | 17.3 | 125 | 15.8 |
| Japan | 83.7 | 26.1 | 74.9 | 21.1 | 55 | 3 |
| Jordan | 74.1 | 19.2 | 65 | 14.8 | 112 | 18.1 |
| Kazakhstan | 70.2 | 17.5 | 63.3 | 14.3 | 198 | 12.6 |
| Kenya | 63.4 | 16.9 | 55.6 | 12.8 | 249 | 51 |
| Kiribati | 66.3 | 16.9 | 58.7 | 12.7 | 198 | 55.9 |
| Kuwait | 74.7 | 17.9 | 65.7 | 13.8 | 81 | 8.7 |
| Kyrgyzstan | 71.1 | 17.9 | 63.9 | 14.6 | 166 | 22.3 |
| Lao People's Democratic Republic | 65.7 | 16.8 | 57.9 | 12.4 | 194 | 66.1 |
| Latvia | 74.6 | 20.1 | 67.1 | 16.5 | 153 | 5 |
| Lebanon | 74.9 | 18.9 | 65.7 | 14.4 | 98 | 8.4 |
| Lesotho | 53.7 | 15.8 | 46.6 | 11.7 | 484 | 97.9 |
| Liberia | 61.4 | 15.6 | 52.7 | 11.2 | 259 | 70.1 |
| Libya | 72.7 | 18.4 | 63.7 | 13.9 | 138 | 13.4 |
| Lithuania | 73.6 | 19.4 | 66.1 | 15.8 | 165 | 5.1 |
| Luxembourg | 82 | 24.3 | 71.8 | 18.9 | 63 | 2.5 |
| Madagascar | 65.5 | 17 | 56.9 | 12.6 | 220 | 48.4 |
| Malawi | 58.3 | 15.4 | 51.2 | 11.6 | 365 | 59.1 |
| Malaysia | 75 | 19.5 | 66.5 | 15 | 123 | 8.2 |
| Maldives | 78.5 | 21.4 | 69.5 | 16.4 | 61 | 9 |
| Mali | 58.2 | 15.9 | 51.1 | 11.8 | 266 | 114.2 |
| Malta | 81.7 | 24.2 | 71.7 | 18.9 | 54 | 6.9 |
| Mauritania | 63.1 | 16.5 | 55.1 | 12.1 | 205 | 83.9 |
| Mauritius | 74.6 | 20.6 | 66.8 | 16.3 | 146 | 14.3 |
| Mexico | 76.7 | 22.4 | 67.4 | 17.1 | 122 | 15 |
| Micronesia | 69.4 | 17.3 | 62.5 | 13.8 | 166 | 34.3 |
| Moldova | 72.1 | 18 | 64.9 | 14.7 | 157 | 16.2 |
| Mongolia | 68.8 | 17.1 | 62.1 | 13.9 | 222 | 18.8 |
| Montenegro | 76.1 | 19.8 | 67.9 | 15.7 | 106 | 4.1 |
| Morocco | 74.3 | 19.4 | 65.1 | 14.8 | 95 | 28 |
| Mozambique | 57.6 | 16.6 | 49.6 | 12 | 355 | 75.1 |
| Myanmar | 66.6 | 16.8 | 59.1 | 12.6 | 199 | 52.7 |
| Namibia | 65.8 | 17.4 | 57.5 | 13.2 | 248 | 48 |
| Nepal | 69.2 | 17.4 | 61.2 | 13.3 | 165 | 36.1 |
| Netherlands | 81.9 | 24.2 | 72.2 | 19.3 | 57 | 3.9 |
| New Zealand | 81.6 | 24.7 | 71.6 | 19.4 | 66 | 5.6 |
| Nicaragua | 74.8 | 21.9 | 63.8 | 15 | 145 | 20.3 |
| Niger | 61.8 | 16.2 | 54.2 | 11.9 | 220 | 95 |
| Nigeria | 54.5 | 14.2 | 47.7 | 10.6 | 344 | 108 |
| North Korea | 70.6 | 17 | 64 | 13.9 | 139 | 21.1 |
| Norway | 81.8 | 24.2 | 72 | 19 | 59 | 2.7 |
| Oman | 76.6 | 20.9 | 66.6 | 15.5 | 99 | 10.9 |
| Pakistan | 66.4 | 17.8 | 57.8 | 13 | 161 | 81 |
| Panama | 77.8 | 24.1 | 68.1 | 18.4 | 118 | 16.9 |
| Papua New Guinea | 62.9 | 15 | 56.4 | 11.6 | 275 | 56.2 |
| Paraguay | 74 | 21.2 | 65.2 | 16.3 | 146 | 20.6 |
| Peru | 75.5 | 21.6 | 65.7 | 16 | 123 | 16 |
| Philippines | 68.5 | 17.1 | 61.1 | 13.3 | 211 | 28 |
| Poland | 77.5 | 21.8 | 68.7 | 17 | 117 | 4.9 |
| Portugal | 81.1 | 24.1 | 71.4 | 19 | 76 | 3.5 |
| Qatar | 78.2 | 21.3 | 67.8 | 15.8 | 68 | 8.6 |
| Romania | 75 | 20.2 | 66.8 | 15.9 | 133 | 9.2 |
| Russia | 70.5 | 18.6 | 63.4 | 15.1 | 222 | 8 |
| Rwanda | 66.1 | 18 | 56.6 | 13.1 | 227 | 40.5 |
| Saint Lucia | 75.2 | 21.3 | 66.1 | 16.3 | 138 | 13.7 |
| Saint Vincent and the Grenadines | 73.2 | 20.1 | 64.6 | 15.4 | 156 | 17.2 |
| Samoa | 74 | 19.2 | 66.6 | 15.3 | 125 | 17.7 |
| Sao Tome and Principe | 67.5 | 18.3 | 59 | 13.7 | 190 | 35.2 |
| Saudi Arabia | 74.5 | 18.7 | 64.4 | 13.8 | 88 | 13.3 |
| Senegal | 66.7 | 16.8 | 58.3 | 12.6 | 188 | 49.5 |
| Serbia | 75.6 | 19.7 | 67.7 | 15.8 | 121 | 6.2 |
| Seychelles | 73.2 | 19.5 | 65.5 | 15.3 | 168 | 14.5 |
| Sierra Leone | 50.1 | 13 | 44.4 | 9.8 | 413 | 118.8 |
| Singapore | 83.1 | 25.5 | 73.9 | 20.2 | 55 | 2.7 |
| Slovakia | 76.7 | 20.7 | 68.1 | 16.2 | 109 | 6 |
| Slovenia | 80.8 | 23.6 | 71.1 | 18.2 | 74 | 2.4 |
| Solomon Islands | 69.2 | 17 | 62.1 | 13.4 | 177 | 26.3 |
| Somalia | 55 | 16.3 | 47.8 | 11.5 | 312 | 136.7 |
| South Africa | 62.9 | 16.6 | 54.4 | 12.3 | 328 | 44.1 |
| South Korea | 82.3 | 25 | 73.2 | 20 | 64 | 3.5 |
| South Sudan | 57.3 | 16.5 | 49.9 | 12.1 | 332 | 93.8 |
| Spain | 82.8 | 25.2 | 72.4 | 19.4 | 56 | 3.4 |
| Sri Lanka | 74.9 | 20.7 | 67 | 16.3 | 138 | 9.6 |
| Sudan | 64.1 | 17.8 | 55.9 | 13.1 | 225 | 67.1 |
| Suriname | 71.6 | 19.3 | 63.1 | 14.7 | 176 | 20.6 |
| Swaziland | 58.9 | 16 | 50.9 | 11.9 | 373 | 71.3 |
| Sweden | 82.4 | 24.6 | 72 | 19.1 | 53 | 2.9 |
| Switzerland | 83.4 | 25.5 | 73.1 | 19.9 | 49 | 4.1 |
| Syrian Arab Republic | 64.5 | 18.1 | 55.9 | 13.1 | 293 | 17.4 |
| Tajikistan | 69.7 | 18.4 | 62.1 | 14.4 | 161 | 44.5 |
| Tanzania | 61.8 | 16.4 | 54.2 | 12.3 | 279 | 58.8 |
| Thailand | 74.9 | 21 | 66.8 | 16.5 | 148 | 12.6 |
| The FYR of Macedonia | 75.7 | 19.1 | 67.5 | 15.1 | 102 | 12 |
| Timor-Leste | 68.3 | 17.1 | 61.1 | 13.3 | 152 | 51.6 |
| Togo | 59.9 | 15.5 | 52.8 | 11.9 | 287 | 78 |
| Tonga | 73.5 | 18.8 | 66 | 15 | 133 | 16.8 |
| Trinidad and Tobago | 71.2 | 18.3 | 63.3 | 14.2 | 170 | 19.1 |
| Tunisia | 75.3 | 19.7 | 66.7 | 15.4 | 100 | 14 |
| Turkey | 75.8 | 21.1 | 66.2 | 15.9 | 106 | 13.6 |
| Turkmenistan | 66.3 | 16.7 | 59.8 | 13.5 | 215 | 52.6 |
| Uganda | 62.3 | 17.3 | 54 | 12.6 | 291 | 55.9 |
| Ukraine | 71.3 | 18.3 | 64.1 | 15 | 195 | 9.4 |
| United Arab Emirates | 77.1 | 20.1 | 68.3 | 15.8 | 75 | 7.8 |
| United Kingdom | 81.2 | 24.1 | 71.4 | 18.8 | 69 | 4.4 |
| USA | 79.3 | 23.6 | 69.1 | 18.1 | 103 | 6.6 |
| Uruguay | 77 | 22 | 67.9 | 17 | 116 | 9.3 |
| Uzbekistan | 69.4 | 18.4 | 62.4 | 14.9 | 184 | 25.8 |
| Vanuatu | 72 | 18.2 | 64.6 | 14.5 | 130 | 28.2 |
| Venezuela | 74.1 | 21.4 | 65.2 | 16.3 | 157 | 16.6 |
| Viet Nam | 76 | 22.5 | 66.6 | 16.8 | 127 | 22 |
| Yemen | 65.7 | 16.4 | 57.7 | 12.2 | 224 | 55.3 |
| Zambia | 61.8 | 17.7 | 53.7 | 13.3 | 303 | 66.1 |
| Zimbabwe | 60.7 | 17.8 | 52.1 | 12.6 | 336 | 59.9 |

LE: Life Expectancy at Birth (in years); LE60: Life Expectancy at the age 60 (in years); HALE: Healthy Life Expectancy at Birth (in years); HALE60: Healthy Life Expectancy at the age 60 (in years); AMR: Adult Mortality Rate (per 100,000 population); U5MR: Under 5 Mortality Rate (per 1000 livebirths).
