# Supplementary material for: Health and economic growth: Evidence from dynamic panel data of 143 years
Source: PLoS One. 2018 Oct 17;13(10):e0204940. doi: 10.1371/journal.pone.0204940 (PMC6192630; doi:10.1371/journal.pone.0204940)
Supplement: S3 Table — Dependent variable is natural logarithm of real GDP per capita at PPP prices for column I-II and growth rate of real GDP per capita (at PPP prices) for column III-IV. The explanatory variables are: INFLATION: difference in natural logarithm of CPI; INVEST: investment to GDP ratio; GOVT_EXP: government expenditure to GDP ratio; LIFE EXPECTANCY: life expectancy at birth; OPEN: total merchandise trade to GDP ratio; SCHOOLING: average number of total years of schooling. Standard errors are heteroscedasticity corrected robust errors and are presented in parentheses. */**/*** denote statistical significance at 10/5/1 percent, respectively. (DOCX) [file pone.0204940.s003.docx]

**Table S3: Pooled OLS results excluding Schooling as explanatory variable**

|  | I | II | III | IV |
| --- | --- | --- | --- | --- |
|  | LogGDP as dependent variable | | Growth as dependent variable | |
| INFLATION | -0.8889** | -1.2794*** | -0.0629* | -0.0675** |
|  | (0.3399) | (0.2808) | (0.0322) | (0.0329) |
| INVEST | -0.8352 | 0.3168 | 0.1306*** | 0.1579*** |
|  | (0.5962) | (0.5568) | (0.0303) | (0.0382) |
| GOVT_EXP | 0.3432*** | 1.2280*** | 0.0019 | -0.0079 |
|  | (0.1906) | (0.2194) | (0.0071) | (0.0101) |
| OPEN | 0.2021*** | 0.4313*** | -0.0043** | -0.0049 |
|  | (0.0614) | (0.1133) | (0.0020) | (0.0038) |
| LIFE EXPECTANCY | 0.0637*** | 0.0501*** | -0.00001 | -0.00006 |
|  | (0.0037) | (0.0037) | (0.0001) | (0.0002) |
| Intercept | 4.6504*** | 5.7208*** | 0.0056 | -0.0038 |
|  | (0.1593) | (0.1381) | (0.0053) | (0.0095) |
| Fixed Effects | No | Yes | No | No |
| No. of Obs. | 185 | 185 | Yes | Yes |
| R^2^ | 0.85 | 0.93 | 0.20 | 0.25 |

Dependent variable is natural logarithm of real GDP per capita (at PPP prices) for column I-II and growth rate of real GDP per capita for column III-IV. The explanatory variables are: INFLATION: difference in natural logarithm of CPI; INVEST: investment to GDP ratio; GOVT_EXP*:* government expenditure to GDP ratio; LIFE EXPECTANCY: life expectancy at birth; OPEN: total merchandise trade to GDP ratio; SCHOOLING: average number of total years of schooling. Standard errors are heteroscedasticity corrected robust errors and are presented in parentheses. */**/*** denote statistical significance at 10/5/1 percent, respectively.
