# Supplementary material for: Health and economic growth: Evidence from dynamic panel data of 143 years
Source: PLoS One. 2018 Oct 17;13(10):e0204940. doi: 10.1371/journal.pone.0204940 (PMC6192630; doi:10.1371/journal.pone.0204940)
Supplement: S4 Table — Dependent variable is natural logarithm of real GDP per capita at PPP prices for column I-II and growth rate of real GDP per capita (at PPP prices) for column III-IV. The explanatory variables are: INFLATION: difference in natural logarithm of CPI; INVEST: investment to GDP ratio; GOVT_EXP: government expenditure to GDP ratio; OPEN: total merchandise trade to GDP ratio; SCHOOLING: average number of total years of schooling. Standard errors are heteroscedasticity corrected robust errors and are presented in parentheses. */**/*** denote statistical significance at 10/5/1 percent, respectively. (DOCX) [file pone.0204940.s004.docx]

**Table S4: Pooled OLS results excluding Life Expectancy as explanatory variable**

|  | I | II | III | IV |
| --- | --- | --- | --- | --- |
|  | LogGDP as dependent variable | | Growth as dependent variable | |
| INFLATION | -0.1326 | -0.1680** | -0.0035 | -0.0035 |
|  | (0.0861) | (0.0761) | (0.0088) | (0.0096) |
| INVEST | 1.3417*** | 2.4457*** | 0.1253*** | 0.1387*** |
|  | (0.4842) | (0.3857) | (0.0233) | (0.0305) |
| GOVT_EXP | 0.7082*** | 0.2508 | 0.0031 | 0.0015 |
|  | (0.1311) | (0.1870) | (0.0056) | (0.0092) |
| OPEN | 0.2089*** | 0.1881*** | -0.0037* | -0.0044 |
|  | (0.0489) | (0.0637) | (0.0019) | (0.0035) |
| Schooling | 0.2098*** | 0.2300*** | -0.0009** | -0.0009* |
|  | (0.0081) | (0.0098) | (0.0004) | (0.0005) |
| Intercept | 6.8033*** | 6.5715*** | 0.0028 | -0.0020 |
|  | (0.0819) | (0.1307) | (0.0032) | (0.0072) |
| Fixed Effects | No | Yes | No | No |
| No. of Obs. | 219 | 219 | 219 | 219 |
| R^2^ | 0.86 | 0.94 | 0.16 | 0.18 |

Dependent variable is natural logarithm of real GDP per capita (at PPP prices) for column I-II and growth rate of real GDP per capita for column III-IV. The explanatory variables are: INFLATION: difference in natural logarithm of CPI; INVEST: investment to GDP ratio; GOVT_EXP*:* government expenditure to GDP ratio; OPEN: total merchandise trade to GDP ratio; SCHOOLING: average number of total years of schooling. Standard errors are heteroscedasticity corrected robust errors and are presented in parentheses. */**/*** denote statistical significance at 10/5/1 percent, respectively.
