# Supplementary material for: Health and economic growth: Evidence from dynamic panel data of 143 years
Source: PLoS One. 2018 Oct 17;13(10):e0204940. doi: 10.1371/journal.pone.0204940 (PMC6192630; doi:10.1371/journal.pone.0204940)
Supplement: S5 Table — Dependent variable is natural logarithm of real GDP per capita at PPP prices for column I-II and growth rate of real GDP per capita (at PPP prices) for column III-IV. The explanatory variables are: INITIAL: natural logarithm of initial real per capita income (at PPP prices); INFLATION: calculated as difference in natural logarithm of CPI; INVEST: investment to GDP ratio; GOVT_EXP: government expenditure to GDP ratio; LIFE EXPECTANCY: life expectancy at birth; OPEN: total merchandise trade to GDP ratio. Standard errors are heteroscedasticity corrected robust errors and are presented in parentheses. */**/*** denote statistical significance at 10/5/1 percent, respectively. Null hypothesis of AR (2) test: errors are serially uncorrelated of order 2. Null hypothesis of Hansen test: there are no overidentifying restrictions. (DOCX) [file pone.0204940.s005.docx]

**Table S5: Panel GMM results excluding Schooling as explanatory variable**

|  | First Difference GMM | System GMM | First Difference GMM | System GMM |
| --- | --- | --- | --- | --- |
|  | LogGDP as Dependent Variable | | Growth as Dependent Variable | |
| $\mathrm{LogGDP}_{t-1}$ | 0.7470*** | 0.7980*** | -0.0362* | -0.0189 |
|  | (0.1091) | (0.0545) | (0.0216) | (0.0132) |
| INFLATION | -0.0033 | 0.0105 | -0.0103 | -0.0066 |
|  | (0.0846) | (0.0930) | (0.0120) | (0.0130) |
| INVEST | 0.7848 | 1.1232*** | 0.0788 | 0.1687*** |
|  | (0.5563) | (0.3215) | (0.0979) | (0.0510) |
| GOVT_EXP | -0.0408 | -0.0188 | 0.0205 | 0.0022 |
|  | (0.2605) | (0.1233) | (0.0399) | (0.0121) |
| OPEN | 0.1341 | 0.0501 | 0.0198 | 0.0038 |
|  | (0.1446) | (0.0406) | (0.0226) | (0.0038) |
| SCHOOLING | 0.0563** | 0.0431*** | 0.0066 | 0.0029 |
|  | (0.0278) | (0.0144) | (0.0056) | (0.0036) |
| No. of Obs. | 200 | 219 | 200 | 219 |
| No. of Instruments  Hansen Test p-value  AR (2) test p-value | 12  0.753  0.296 | 19  0.519  0.254 | 12  0.130  0.997 | 19  0.268  0.780 |

Dependent variable is natural logarithm of real GDP per capita (at PPP prices) for column I-II and growth rate of real GDP per capita (at PPP) for column III-IV. The explanatory variables are: INITIAL: natural logarithm of initial real per capita income; INFLATION: is calculated as difference in natural logarithm of CPI; INVEST: investment to GDP ratio; GOVT_EXP: government expenditure to GDP ratio; LIFE EXPECTANCY: life expectancy at birth; OPEN: total merchandise trade to GDP ratio. Standard errors are heteroscedasticity corrected robust errors and presented in parentheses. */**/*** denote statistical significance at 10/5/1 percent respectively. Null hypothesis of AR (2) test: errors are serially uncorrelated at order 2. Null hypothesis of Hansen test: there are no overidentifying restrictions.
